# Localized Provoked Vulvodynia-An Ignored Vulvar Pain Syndrome

**DOI:** 10.3389/fcimb.2021.678961

**Published:** 2021-06-17

**Authors:** Jorma Paavonen, David A. Eschenbach

**Affiliations:** ^1^ Faculty of Medicine, University of Helsinki, Helsinki, Finland; ^2^ Department of Obstetrics and Gynecology, University of Washington, Women’s Health Care Center- Roosevelt, Seattle, WA, United States

**Keywords:** localized provoked vulvodynia, vulvar pain syndrome, vulvar vestibulitis syndrome, vulvodynia, vulvar pain

## Abstract

Localized provoked vulvodynia (LPV) causes dyspareunia among reproductive aged women. We review the pathogenesis of LPV and suggest that LPV is an inflammatory pain syndrome of the vestibular mucosa triggered by microbial antigens in a susceptible host. Tissue inflammation and hyperinnervation are characteristic findings which explain symptoms and clinical signs. Education of health care providers of LPV is important since this condition is common, often unrecognized, and patients often become frustrated users of health care. Research is needed on the antigen triggers of the syndrome. Randomized clinical trials are needed to evaluate treatment modalities.

## Highlights

Localized provoked vulvodynia is a major cause of dyspareunia and is triggered by inflammation and hyperinnervation of the vestibular mucosa. Education of health care providers is important since LPV is common, but often remains unrecognized.

## Introduction

Localized provoked vulvodynia (LPV) is defined as vulvar pain induced by touching the vulvar vestibular epithelium that has lasted a minimum of 3 months, in the absence of another recognizable vulvar disease (American College of Obstetricians and Gynecologists’ Committee on Gynecologic Practice and American Society for Colposcopy and Cervical Pathology (ASCCP), 2016; Bornstein et al., 2016). The onset can be primary (present at the first attempted sexual intercourse) or secondary appearing later in life after a period of painless sexual intercourse. The dyspareunia associated with LPV causes sexual dysfunction, and severely impacts sexual, mental and social health. The prevalence of LPV varies between 8 and 18% in population based studies (Harlow et al., 2014). LPV is a poorly recognized condition. For instance, in one study only 50% of symptomatic women sought care, 30% had three or more physician visits, and 40% remained undiagnosed (Harlow et al., 2001). The term localized provoked vulvodynia (LPV) has been universally accepted and holds priority over the former term vulvar vestibulitis syndrome (Bornstein et al., 2016).

## Diagnosis

**Figure 1:**
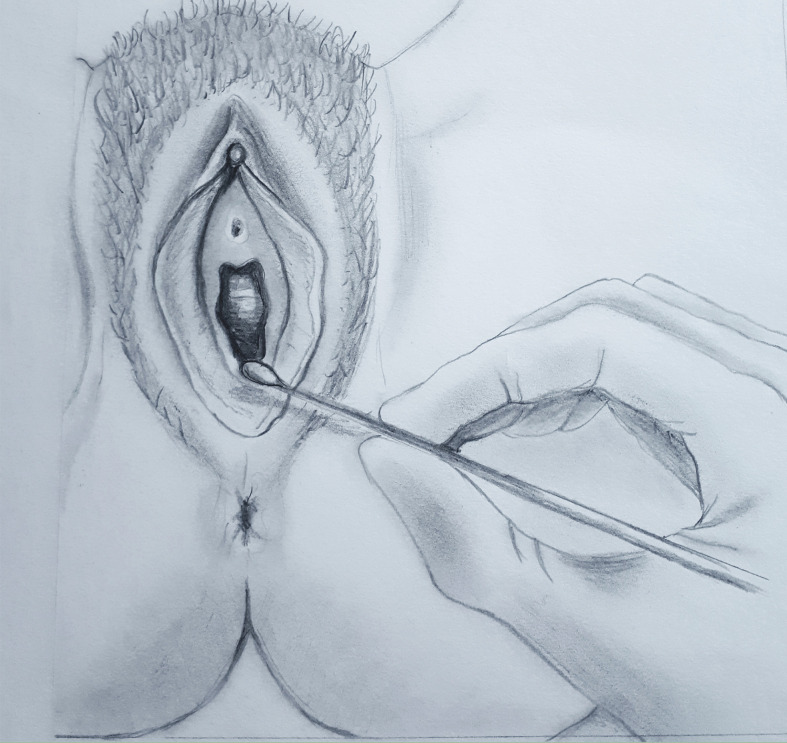
Q-tip test. Vestibular point tenderness is tested at 2-, 5-, 6-, 7-, and 10-o’clock positions, and quantified as mild, moderate, or severe.

The clinical diagnostic test is known as the Q-tip test and is performed using a simple cotton swab gently touching the vestibular epithelium (**Figure 1**) (American College of Obstetricians and Gynecologists’ Committee on Gynecologic Practice and American Society for Colposcopy and Cervical Pathology (ASCCP), 2016; Bornstein et al., 2016). The most characteristic painful areas are around the ductal openings of the vestibular glands, most commonly around the posteriorly located Bartholin’s glands and less commonly around the anteriorly located Skene’s glands (Goldstein et al., 2016). Patients with LPV in the anterior vestibule often have external dysuria that should be differentiated from cystitis or urethritis. Cervicovaginal infections and other gynecological or dermatological conditions causing vulvar pain should be excluded. Dermatoses such as lichen sclerosus, lichen planus or lichen simplex chronicus can usually be diagnosed by their characteristic appearance or by biopsy.

## Pathogenesis

The pathogenesis of LPV involves microbial, immunological and genetic components. Inflammation and hyperesthesia within well-defined areas of the vestibule well explain the symptoms and signs of LPV (Tommola et al., 2015; Tommola et al., 2016). Many lines of evidence suggest that Candida is one trigger antigen causing LPV. Women with LPV have a history of recurrent vulvovaginal candidiasis (RVVC) more often than control women. This finding has been consistently replicated in several case-control studies of LPV (Leusink et al., 2018). Further, women with LPV often have cutaneous hypersensitivity to *Candida albicans* as detected by patch skin testing. Ramirez De Knott et al. reported that 10 of 27 LPV patients tested positive for *C. albicans* cutaneous hypersensitivity compared to 0 of 13 control women (p=0.01) (Ramirez De Knott et al., 2005). A familiarity analysis of LPV patients treated by vestibulectomy suggests a genetic predisposition (Morgan et al., 2016). A mouse model of repeated vulvovaginal *C. albicans* infections replicates the clinical findings of vulvodynia and its characteristic histopathological changes (Farmer et al., 2011). *In vitro* studies also demonstrate that vestibular fibroblasts from LPV patients produce more proinflammatory cytokines such as interleukin (IL)-1 beta, IL-6 and IL-8, as well as prostaglandin E2 when stimulated with *Candida* antigens than fibroblasts from control women (Foster et al., 2007; Foster et al., 2015). Fibroblasts from LPV patients expressed higher levels of dectin-1 fungal antigen receptor and recognized lower concentrations of *Candida* antigen than fibroblasts from control subjects (Falsetta et al., 2015).

**Figure 2:**
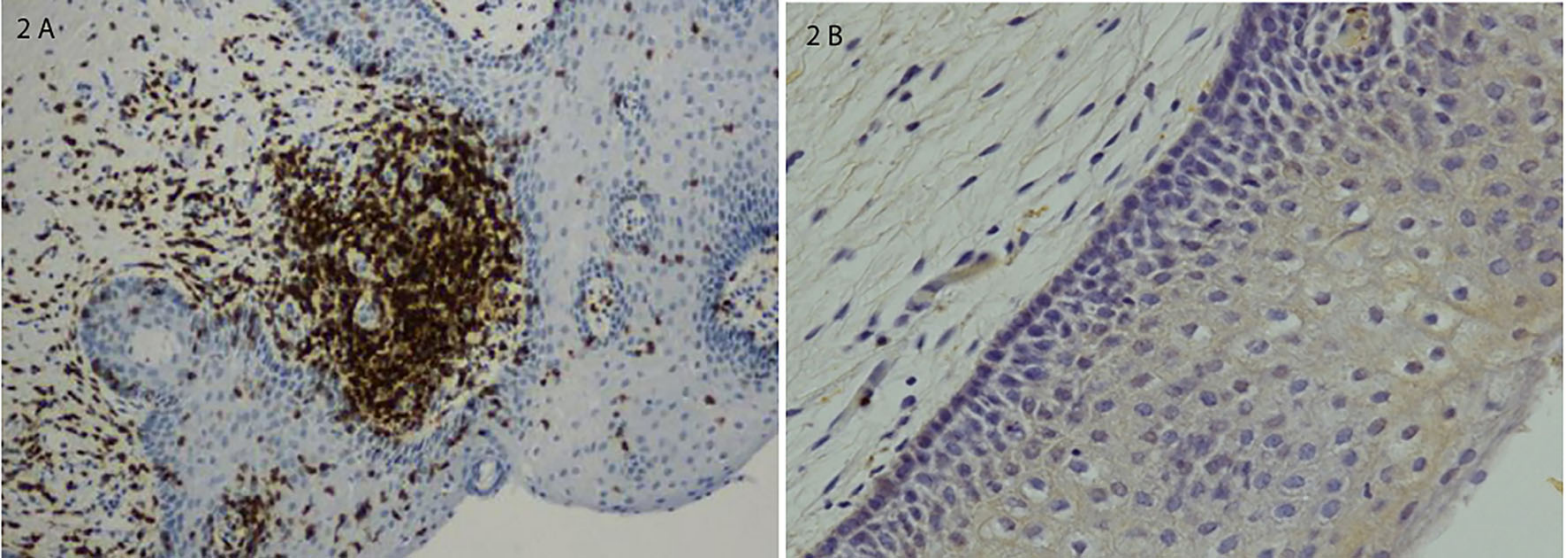
**(A)** Lymphoid aggregate in a vestibular biopsy from a patient with severe LPV; immunostaining for T cells and B cells. **(B)** Normal vestibular epithelium without inflammatory changes. Histological sections were counterstained with hematoxylin and photomicrographed using x10 objective. Adapted from Tommola P., et al., Am J Obstet Gynecol7; 2015;212(4):476.e1-8 (by permission).

Other less extensively studied trigger antigens may also play a role in the pathogenesis of LPV. The histopathology of the vulvar vestibule in LPV demonstrates two distinctive pathologic features, infiltration with lymphocytic inflammatory cells and proliferation of sensory nerve fibers. Several studies report chronic inflammation in LPV (Halperin et al., 2005; Goetsch et al., 2010). Increased CD4-positive T helper cell densities (Leclair et al., 2014) and elevated tissue levels of IL-1 beta and tumor necrosis factor-alpha (TNF-α) were found (Foster and Hasday, 1997). In areas with the highest densities of inflammation, T and B cells form lymphoid aggregates that represent secondary lymphoid tissue (Tommola et al., 2015; **Figures 2A, B**). The presence of lymphoid aggregates and increased density of B cells, including activated plasma cells are the major differences between the vestibular tissue from women with LPV and samples from control women (Leusink et al., 2018). Overall, the total number of inflammatory cells is markedly increased in the tender areas of the vestibule (Tommola et al., 2015). The first reports on neural tissue changes in LPV originated from immunohistochemistry studies that reported more intraepithelial nerve fibers in LPV than in controls (Bohm-Starke et al., 1998; Bohm-Starke et al., 1999). Nerve fiber proliferation and hyperinnervation is consistently observed in virtually all histopathological studies of the vestibular areas of LPV patients. The density of intraepithelial nerve fibers was higher in vestibular samples with lymphoid aggregates than in vestibular samples without lymphoid aggregates (Tommola et al., 2016). Glandular epithelium also showed proliferation of epithelial nerve fibers with higher densities in glands surrounded by B cell infiltrates than in glands without such infiltrates. This hyperinnervation is derived by sprouting sensory axons (Liao et al., 2017). Thus, tender zone hyperinnervation is due at least in part to proliferation of nerve fibers known to detect mechanical pain contributing to the hypersensitivity to touch in LPV. (Liao et al. (2017) demonstrated that inflammatory hypersensitivity and hyperinnervation also occur in concert with a local renin-angiotensin system (RAS), and that tender tissue shows a markedly robust RAS.

In aggregate, the pathophysiological evidence suggests that LPV has a biological basis and that microbial antigens such as Candida can induce a prolonged cutaneous hypersensitivity response in the vulvar vestibular epithelial cells in a subset of susceptible women. However, the overall evidence is still relatively limited, and some studies question the role of inflammation in driving LPV (Chalmers et al., 2016). Stress-induced life factors (Harlow and Gunther Stewart, 2005; Khandker et al., 2011; Khandker et al., 2014; Khandker et al., 2019) can also trigger an immunological tissue response. The pathogenesis of primary LPV and secondary LPV may also differ so that the microbial cause is more likely related to secondary LPV. Clearly, LPV is a clinical diagnosis and may have multiple etiologies.

## Management

Recent reviews of the management of LPV (Spoelstra et al., 2013; Goldstein et al., 2016; Miranda Varella Pereira et al., 2018) have concluded that most studies are underpowered and fail to demonstrate beneficial effects of an intervention. An expert committee reviewed the evidence on the treatment of women’s vulvar pain syndrome and recommended the following treatments for the management of LPV: psychological interventions, pelvic floor physical therapy, and vestibulectomy (Goldstein et al., 2016). Psychological interventions, such as cognitive behavioral therapy or psychosexual counseling, may help patients to cope with the symptoms and reduce central pain hypersensitivity. In a randomized study, cognitive behavioral therapy proved more effective in LPV than traditional support or topical corticosteroids (Bergeron et al., 2016). Biofeedback therapy and myofascial trigger point manipulation reduces pelvic floor muscle spasm and dysfunction and normalizes muscle tone by pain desensitization. Physical therapy and psychosexual counseling are often combined. However, most studies evaluating these therapeutic approaches lack comparison groups. Botulinum toxin has been used to decrease peripheral and central sensitization and pelvic floor dysfunction associated with LPV. Although case-series and case reports have demonstrated efficacy, botulinum toxin is not recommended as first-line treatment for LPV, pending more robust scientific support  (Goldstein et al., 2016).

Surgical treatment of LPV consists of removal of the posterior vestibular epithelium by a vestibulectomy. In clinical practice, surgery has been only recommended for patients with severe LPV refractory to conservative treatment (Tommola et al., 2010; American College of Obstetricians and Gynecologists’ Committee on Gynecologic Practice and American Society for Colposcopy and Cervical Pathology (ASCCP), 2016; Goldstein et al., 2016). Long-term patient satisfaction after surgery can be explained by the mucosal pathophysiology since surgery removes the localized tender mucosa superficially and replaces it with advanced vaginal mucosa. Overall, 80-90% of the patients reported a partial or complete response (Tommola et al., 2010; Tommola and Unkila-Kallio Paavonen, 2011). However, randomized trials of the effectiveness of surgery are needed. **Table 1** shows a summary of selected LPV treatment modalities by strength of evidence.

**Table 1:** Selected treatment modalities for localised provoked vulvodynia (LPV) by quality of evidence.

Treatment modality	Quality of evidence/Code	Recommendation
Psychological intervention such as cognitive behavioural therapy	High/A	Recommended
Biofeedback therapy with myofascial trigger point manipulation	High/A	Recommended
Botulinum toxin injections	Low/C	Not recommended
Topical corticosteroid creams	Very low/D	Not recommended
Local corticosteroid injections*	Very low/D	Not recommended
Discontinuation of combined oral contraceptives	Very low/D	Recommended#
Oral antimycotics**	Moderate/B	Recommended
Oral amitriptyline or other drugs used for neuropathic pain	Moderate/B	Not recommended
Vestibulectomy***	Moderate/B	Recommended

*Submucosally into vestibular trigger points; **Fluconazole 150mg weekly as maintenance therapy; ***Evidence based on treatment of refractory cases; RCTs pending; #RCTs pending.

**Figure 3:**
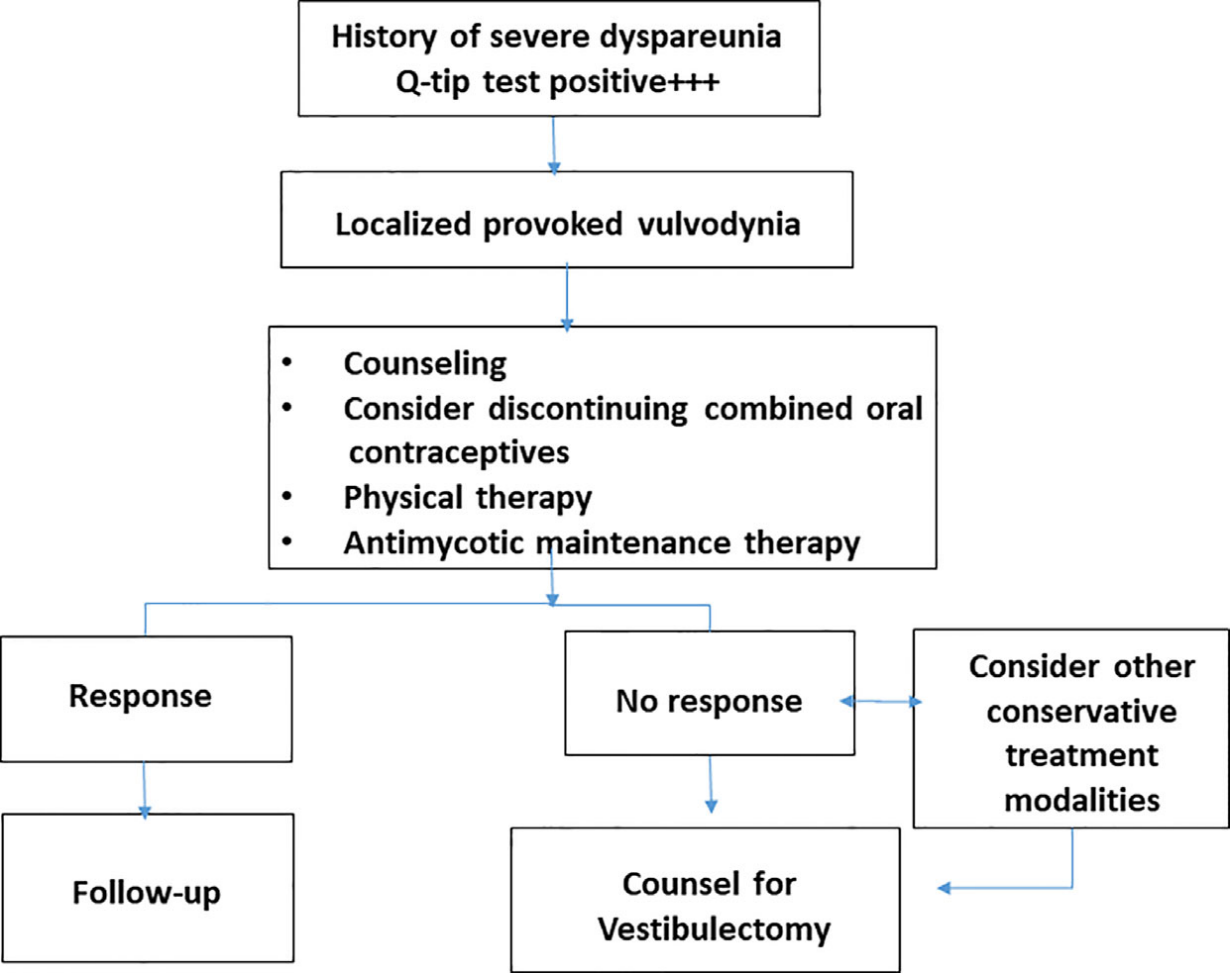
Clinical algorithm developed by the authors and recommended for the management of patients with severe vulvar vestibulitis syndrome.

A useful clinical management algorithm has been developed by the American College of Obstetricians and Gynecologists and the American Society for Colposcopy and Cervical Pathology (**Figure 3**) (American College of Obstetricians and Gynecologists’ Committee on Gynecologic Practice and American Society for Colposcopy and Cervical Pathology (ASCCP), 2016). One additional step in the management is to encourage women to discontinue the use of combined oral contraceptives. This is based on expert opinion and clinical experience of alleviation of allodynia with return of natural menstrual cycle. Women using oral contraceptives may have decreased mechanical pain threshold in the vestibular mucosa (Bohm-Starke et al., 2004). However, without better evidence this recommandation is not universally accepted.

## Conclusions

In conclusion, the landmark histopathologic findings of chronic inflammation and hyperinnervation in LPV well explain the clinical symptoms and signs. Inflammation in the vestibular tissue and its association with hyperinnervation derived by mechanical nociceptor axons results in the altered pain sensation and hypersensitivity. The vestibular mucosa is a key mucocutaneous surface with an important role in immune responses in general (Robert and Kupper, 1999). Education of health care providers on the diagnosis and management of LPV is important, since such patients often are frustrated users of the health care system.

## Author Contributions

The authors report equal roles in the conception, planning, carrying out, and writing up the work. All authors contributed to the article and approved the submitted version.

## Conflict of Interest

The authors declare that the research was conducted in the absence of any commercial or financial relationships that could be construed as a potential conflict of interest.
